# Construction of a sensitive indicator evaluation system for sepsis care quality: a three-stage mixed-method Delphi study

**DOI:** 10.3389/fmed.2026.1815560

**Published:** 2026-05-04

**Authors:** Chen Huang, Huan Liu, Wenwen Qi, Shuyuan Zhao, Jinghua Xu, Feng Jing, Erzhen Chen

**Affiliations:** 1 Department of Nursing, Ruijin Hospital, Shanghai Jiao Tong University School of Medicine, Shanghai, China; 2 Department of Emergency, Ruijin Hospital, Shanghai Jiao Tong University School of Medicine, Shanghai, China; 3 Shanghai Institute of Aviation Medicine, Ruijin Hospital, Shanghai Jiao Tong University School of Medicine, Shanghai, China; 4 Emergency and Critical Care Medicine Center, Ruijin Hospital, Shanghai Jiao Tong University School of Medicine, Shanghai, China

**Keywords:** Delphi method, mixed-methods research, nursing-sensitive quality indicator, quality indicator system, sepsis management

## Abstract

**Background:**

The development of care-sensitive quality indicators represents an essential component of care management. Currently, there exists no objective, scientific, and sensitive assessment framework for evaluating sepsis care quality management in China.

**Methods and design:**

This mixed-methods investigation employed a three-phase design. Initially, a systematic literature review (2014–2024) across four databases identified evidence regarding sepsis care quality. Subsequently, semi-structured interviews were conducted with five clinical experts from emergency departments and intensive care units (ICUs) to examine their perspectives on nursing-sensitive quality indicators (NSQIs). Finally, a modified Delphi process engaged a multidisciplinary panel to refine and validate sepsis-specific NSQIs through systematic consensus-building.

**Results:**

Two rounds of expert consultation were completed with a questionnaire return rate of 100%. Sixteen experts, consisting of 10 nurses and 6 physicians, participated in the first and second rounds of the Delphi survey, respectively. The mean score of the expert authority coefficient Cr for the two rounds was 0.95 and 0.96 (Cr ≥ 0.7). The coordination coefficient (Kendall W) was 0.120-0.316 (*p* < 0.001) in the first round and 0.116–0.142 (*p* < 0.001) in the second round, both of which reached a significant consensus. A comprehensive list of sensitive indicators for sepsis care quality was established, encompassing three primary, nine secondary, and 30 tertiary indicators.

**Conclusion:**

The established NSQIs encompass three fundamental dimensions of sepsis care quality: importance, rationality, and feasibility.

**Clinical practice implications:**

This research provides a valuable framework for evaluating clinical care quality in sepsis management.

## Introduction

Sepsis represents a critical global health challenge (1). It manifests as life-threatening organ dysfunction caused by a dysregulated immune response to infection (2). The condition’s time-sensitive nature requires prompt clinical intervention to minimize mortality and morbidity (3, 4). The Surviving Sepsis Campaign (SSC) has continuously updated evidence-based sepsis management protocols over the years, with the latest 2021 international guidelines reinforcing the core of the 1-h bundle and emphasizing individualized optimization of intervention strategies; consistent implementation of these guidelines has been proven to significantly enhance clinical prognosis in sepsis patients (5, 6). As frontline healthcare providers, nurses are essential in early sepsis recognition and protocol-driven management (6, 7). Patient outcomes in sepsis cases correlate directly with the quality of care provided.

The Nursing Sensitive Quality Indicator (NSQI) is an essential instrument for care quality assessment (8, 9). China’s healthcare system features distinct characteristics that necessitate a tailored sepsis care quality indicator framework: the nurse-to-patient ratio in clinical settings such as emergency departments and ICUs differs from that in Western countries, with relatively high nursing workloads in tertiary hospitals; medical resource distribution shows regional disparities, with uneven access to standardized sepsis management protocols between urban and rural medical institutions; meanwhile, the nursing scope of practice in China has clear clinical positioning, with nurses undertaking unique core responsibilities in sepsis early identification, continuous monitoring and protocol-based intervention execution in the local healthcare context. These unique contextual factors mean that internationally developed quality indicators cannot be directly applied to Chinese clinical settings, making the construction of a China-specific sepsis nursing-sensitive quality indicator system an urgent clinical and managerial need.

Grounded in a constructivist paradigm, this study adopted a sequential mixed-methods approach to design a Delphi study, integrating the latest evidence-based research findings and frontline clinical practical insights. It aims to develop a context-adaptive sensitive indicator evaluation system for sepsis care quality suitable for Chinese healthcare settings, and further clarify the specific contribution of NSQIs to optimizing sepsis care processes, standardizing nursing practice, and improving the overall quality of sepsis care. Ultimately, this research strives to provide a scientific and measurable tool for clinical nursing quality assessment and continuous improvement in sepsis management, while offering empirical evidence to support relevant healthcare policy reform and the implementation of international sepsis management guidelines in local clinical practice.

## Background/justification for study

Sepsis is defined as life-threatening organ dysfunction caused by an uncontrolled immune response to infection. It represents a significant global health challenge, accounting for 19.7% of annual deaths worldwide (2, 6). Common symptoms of sepsis include drowsiness, chills or fever, hypothermia, nausea, low blood pressure, and a rapid heartbeat (3). Patients with sepsis may also experience shock, multiple organ failure, decreased urine output, and acute respiratory distress, all of which can lead to death if the condition worsens (2).

The management of sepsis requires comprehensive care, emphasizing early identification and intervention for infections, hemodynamic instability, and multi-organ dysfunction (10). Clinical assessment tools facilitate timely diagnosis, including the Quick Sequential Organ Failure Assessment (qSOFA), Sequential Organ Failure Assessment (SOFA) (11), Systemic Inflammatory Response Syndrome (SIRS) criteria and National Early Warning Score (NEWS) (12, 13) for risk stratification (11, 14). Contemporary guidelines emphasize standardized protocols for early management, including the Surviving Sepsis Campaign (SSC) 1-h bundle (15), which requires lactate measurement, blood culture collection, broad-spectrum antibiotic administration, and crystalloid resuscitation for hypotension or hyperlactatemia (16, 17), alongside the “Sepsis Six” strategy incorporating urine output monitoring (18). Substantial evidence confirms that rapid recognition and adherence to these evidence-based interventions significantly reduce mortality and improve clinical outcomes in sepsis and septic shock (3, 6, 19, 20).

As frontline responders, emergency department (ED) nurses serve a critical function in early sepsis recognition and standardized protocol implementation (2, 6). High-quality care delivery enhances early sepsis detection rates and timely intervention execution (17), reducing adverse events and improving patient outcomes (21). Consequently, identifying reliable and sensitive healthcare quality and safety indicators remains essential for healthcare system improvement (22).

Nursing-sensitive quality indicators (NSQIs) are defined as surrogate measures reflecting the quality and performance of nursing care, encompassing principles, procedures, and scales to quantify care outcomes influenced by nursing structures, processes, and outcomes (23, 24). Based on Donabedian’s structure-process-outcome model (25, 26), NSQIs have evolved as critical tools for evaluating nursing quality, with standardized data collection systems established in high-income countries (e.g., the U.S., Australia) to guide quality improvement initiatives (27, 28). While China initiated NSQI development later than Western counterparts, recent studies demonstrate growing research interest in adapting NSQIs to local contexts (8, 29, 30). However, a significant gap persists: no standardized NSQIs specific to sepsis care have been established in China, which limits the ability to evaluate the impact of nursing on sepsis outcomes. This study aims to address this gap by developing and validating NSQIs tailored to sepsis management in Chinese clinical settings.

## Methodology/design

This study employs a constructivist paradigm, emphasizing the co-construction of knowledge through interactions with clinical experts and evidence synthesis, to address the gap in sepsis-specific NSQIs within the Chinese healthcare context. The methodological framework utilizes a sequential mixed-methods approach, incorporating qualitative exploration and quantitative consensus-building techniques.

### Method

#### Step 1: literature review

**FIGURE 1:**
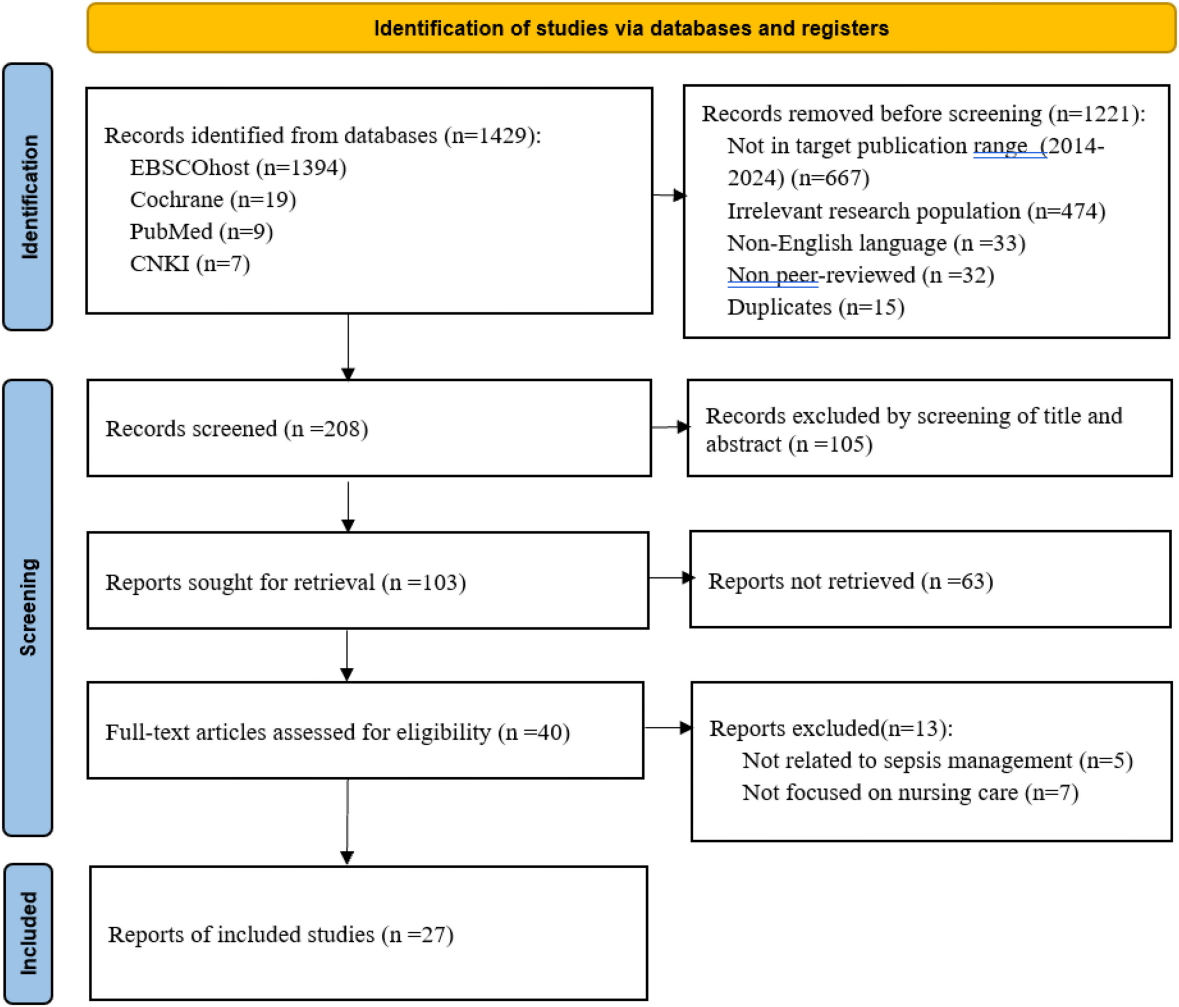
PRISMA for details (Haddaway et al., 2022).

A systematic literature search was conducted across four databases: EBSCOhost, Cochrane, PubMed and China National Knowledge Infrastructure (CNKI). The search spanned from 2014 to 2024, with the unified search strategy constructed using MeSH terms (“sepsis,” “nursing,” “quality indicators”) and free-text keywords (sepsis care, nursing-sensitive quality indicators, sepsis quality management) combined with Boolean operators (AND/OR); database filters were applied for publication year (2014–2024), study type (full-text, peer-reviewed) and research subject (nursing care for sepsis). For English-language databases (EBSCOhost, Cochrane, PubMed), the language filter was set to English; for CNKI, the language filter was set to Chinese, with no English-language restriction applied for this Chinese database. The search aimed to identify studies involving registered nurses or nurse leadership teams focusing on sepsis and quality of care. The inclusion criteria specified that articles must be closely related to sepsis care, full-text, peer-reviewed, and in the corresponding official language of the database (Chinese for CNKI, English for English-language databases) within the specified timeframe.. High-quality Chinese core literature on sepsis nursing and quality management from CNKI was fully included to ensure the contextual adaptability of the indicator system for the Chinese healthcare setting, and English literature was incorporated to align with international evidence-based sepsis management guidelines. Articles were excluded if they involved non-nursing staff, were conference abstracts, did not meet the database-specific language requirements, were not in English, were not peer-reviewed or were inaccessible (see Figure 1, PRISMA for details). All criteria for publication year, language, peer-review status and research population were pre-implemented as database filters, and the large number of records removed prior to screening were the direct results of these pre-set database filterings rather than manual screening. Two trained researchers independently conducted this process, with a third researcher arbitrating any disagreements.

#### Step 2: qualitative phase—semi-structured interviews

The second phase comprised semi-structured interviews with clinical experts specializing in sepsis care from emergency departments and tertiary hospitals’ intensive care units (ICUs). For this study topic, an interview outline was developed (see Supplementary File 1) guided by the symptom management model (32) to explore participants’ views on the sepsis NSQI and the application of symptom management principles in clinical practice. Key topics encompassed symptom recognition, utilization of screening tools, challenges in atypical symptom presentation, critical nursing interventions, interdisciplinary collaboration barriers, and outcome evaluation metrics. Each interview was conducted face-to-face, with an average duration of 45–60 min; data collection was stopped when no new themes or insights emerged from the interviews, confirming that the qualitative data had reached theoretical data saturation. Interviews underwent audio recording, verbatim transcription, and thematic analysis (33) to identify themes related to structural, procedural, and outcome dimensions of sepsis care. These findings informed the initial draft of sepsis-specific NSQIs, subsequently refined through the Delphi consensus process.

#### Step 3: Delphi phase—consensus building and interaction analysis

Building upon the literature review and qualitative interview findings, the third phase implemented a modified Delphi method to transform emerging themes into consensus-based priorities (34). A multidisciplinary panel of 16 experts participated in iterative, anonymous questionnaires to evaluate the importance, rationality of formulation, and operational feasibility of candidate NSQIs for sepsis care. Each round incorporated quantitative ratings (using a 5-point Likert scale) and qualitative feedback to refine the indicators (35). The Delphi process maintained anonymity to minimize hierarchical bias and ensure equitable participation (36). Indicators not meeting consensus thresholds underwent revision or exclusion, yielding a validated set of sepsis-specific NSQIs aligned with clinical priorities and measurable outcomes.

#### Setting and sample

The study employed purposive sampling across two phases. For the semi-structured interviews, five clinical experts—three nurses and two physicians—with extensive sepsis care experience were recruited from emergency departments and ICUs of tertiary hospitals in China. Eligibility criteria for expert participation in the interview phase included: (1) having at least 5 years of clinical practice experience in sepsis care or critical care; (2) holding a professional title of intermediate or above; (3) being familiar with clinical sepsis management protocols and nursing-sensitive quality indicators. The Delphi consensus phase involved a multidisciplinary panel of 16 experts from three Chinese provinces, comprising 10 nursing specialists and 6 medical experts. Eligibility criteria for Delphi panelists were explicitly defined as: (1) having at least 10 years of clinical practice experience in critical care or sepsis management; (2) holding a professional title of senior or above, or having a doctoral academic background; (3) being familiar with sepsis clinical management guidelines and nursing quality indicator development; (4) having participated in sepsis care quality management or clinical indicator research-related work. Delphi panelists represented diverse healthcare settings, including academic medical centers and regional hospitals, ensuring representation of varied clinical practices and institutional contexts. All 16 experts completed both rounds of the Delphi consultation with no dropouts, and all participated in the full process of indicator evaluation and revision.

### Data analysis

The qualitative data from semi-structured interviews underwent analysis using reflexive thematic analysis (RTA) (33). This iterative process emphasizes dynamic engagement between researchers, data, and theoretical frameworks. Following RTA’s six-phase approach, audio recordings underwent verbatim transcription and systematic analysis through repeated immersion in the transcripts to generate codes, construct themes, and refine interpretations (37, 38). Codes derived from participants’ descriptions of clinical challenges were mapped to the Symptom Management Model domains. Potential themes underwent critical review against raw data and contextualization within sepsis care dimensions (structural, procedural, and outcome), with reflexive discussions resolving ambiguities. The final themes informed sepsis-specific NSQIs development.

The Delphi method was employed to develop sensitive indicators for evaluating sepsis care quality. A questionnaire was distributed to field experts, and the data analysis was conducted using EXCEL 2019 and SPSS 26.0 software. The expert questionnaire evaluated each index based on importance, reasonableness, and feasibility using a 5-point scale, with higher scores indicating greater expert recognition. Three criteria were established for entry retention: a mean score of 3.50 or higher, a full score ratio exceeding 20%, and a coefficient of variation below 0.25. Meeting all three criteria indicated strong expert consensus and recognition for that entry, confirming its significance within the system. In addition, the Analytic Hierarchy Process (AHP) was adopted to calculate the weights of the primary, secondary and tertiary indicators of the sepsis care quality sensitive indicator system, with the expert scoring data from the Delphi rounds as the core basis; the consistency of the judgment matrix was tested to ensure the scientific and rationality of the weight assignment, and the combined weights of each indicator were further derived to determine the hierarchical importance of the indicators.

## Results

Following Donabedian’s “Structure-Process-Outcome” model and symptom management theory, a three-tiered framework for sepsis nursing care quality indicators was established through a systematic literature review (encompassing 27 studies, following PRISMA 2020 guidelines) and thematic analysis of semi-structured interviews. The systematic literature review synthesized core findings from the 27 included studies, identifying three core domains of sepsis nursing-sensitive quality indicators consistent with Donabedian’s model (structural, procedural, outcome) and 18 preliminary evidence-based indicators; key themes extracted included early sepsis identification efficiency, compliance with SSC bundle interventions, nursing monitoring standardization, multidisciplinary collaboration effectiveness, and nursing-related clinical outcome evaluation. These domain and indicator findings formed the initial empirical basis for the preliminary indicator pool of the study. The review identified key quality-sensitive domains, while interviews with frontline clinicians revealed three interconnected dimensions: structural, procedural, and outcome-related. The interviews also uncovered implementation barriers, including delayed recognition of atypical symptoms and inconsistent 1-h bundle execution, alongside facilitators such as protocol standardization and real-time decision support systems. These insights were synthesized to refine the preliminary framework into a Delphi consultation scale comprising 3 domains, 7 categories, and 23 evidence-based indicators, ensuring coherence between empirical evidence and clinical practice.

During the Delphi phase, a team of experienced medical and nursing specialists was assembled from five hospitals across three Chinese provinces. The group included 16 experts: 10 nursing professionals and 6 medical specialists. Two rounds of Delphi consultation were conducted to evaluate the importance, rationale, and feasibility of sepsis care sensitivity indicators, leading to a consensus.

In this study, 16 questionnaires were distributed to relevant field experts during both consultation rounds. Table 1 presents the experts’ basic information. All 16 questionnaires were valid, achieving a 100% validity rate for both rounds (see Table 2). In the first round, the coefficient of experts’ judgmental basis (Ca) was 0.98, with the coefficient of experts’ familiarity (Cs) at 0.92, yielding an authority coefficient of 0.95. In the second round, the judgmental basis coefficient remained at 0.98, while the familiarity coefficient (Cs) increased to 0.94, resulting in an authority coefficient of 0.96 (see Table 3).

**TABLE 1:** Demographic characteristics of Delphi panelists.

Variant	Categories	Numbers	Percentage (%)
Age	≤40 years	6	37.50
	41–50 years	6	37.50
	≥51 years	4	25.00
Educational attainment	Undergraduate	7	43.75
	Master’s degree	3	18.75
	Doctoral degree	6	37.50
Professional roles	Postdoctoral fellow	1	6.25
	Physician	5	31.25
	Director of nursing	1	6.25
	Head nurse	4	25.00
	Nurse manager	4	25.00
	Specialist nurse	1	6.25
Primary specialization	Nursing	10	62.50
	Medicine	6	37.50
Clinical experience (acute and critical care)	≤10 years	2	12.50
	11∼20 years	5	31.25
	≥21 years	9	56.25

**TABLE 2:** Response rates of Delphi questionnaire rounds.

Round	Total distributed	Valid responses	Response rate (%)
First round	16	16	100.00
Second round	16	16	100.00

**TABLE 3:** Expert authority coefficients.

Round	Coefficient of experts’ judgmental basis (Ca)	Coefficient of experts’ familiarity (Cs)	Coefficient of experts’ authority
First round	0.98	0.92	0.95
Second round	0.98	0.94	0.96

Expert opinion harmonization was measured using Kendall’s W coefficient to assess inter-expert consistency. The first round analysis of 16 experts’ indicator scores yielded Kendall’s coefficient of concordance (W) values of 0.120, 0.316, and 0.146 for applicability, importance, and operational feasibility, respectively (χ^2^ = 61.429, df = 32, *P* = 0.001; χ^2^ = 161.963, df = 32, P < 0.001; χ^2^ = 74.899, df = 32, P < 0.001). The second round produced Kendall’s W values of 0.116, 0.125, and 0.142 for the three dimensions, respectively (χ^2^ = 75.945, df = 41, *P* = 0.001; χ^2^ = 81.773, df = 41, P < 0.001; χ^2^ = 93.016, df = 41, *P* < 0.001). All indicators in both rounds achieved statistically significant consensus (P < 0.05), with the coefficients of variation (CV) of all indicators falling within the range of 0.07–0.23 in the first round and 0.07–0.24 in the second round. All core consensus statistics including mean scores, CV and Kendall’s W for each round are systematically presented in Table 4 to facilitate the evaluation of expert agreement.

**TABLE 4:** Coefficient of harmonization of expert opinions.

Round	Evaluation dimension	Coordination factor (W)	Chi-square value (χ^2^)	Degrees of freedom (df)	Significance (P)
First round	Applicability	0.120	61.429	32	0.001
	Importance	0.316	161.963	32	<0.001
	Operational feasibility	0.146	74.899	32	<0.001
Second round	Applicability	0.116	75.945	41	0.001
	Importance	0.125	81.773	41	<0.001
	Operational feasibility	0.142	93.016	41	<0.001

### First round Delphi results

The initial evaluation of primary indicators demonstrated strong expert consensus validity. Appropriate scores ranged from 4.81 to 4.88 (CV = 0.07-0.11; full score rate = 87.50%). Importance scores reflected stronger consensus (mean = 4.81–5.00, CV = 0.00–0.11) with full score rates between 87.50 and 100%. Operational feasibility scores matched appropriateness metrics (mean = 4.81–4.88, CV = 0.10–0.11; full score rate = 87.50–93.75%). All primary indicators satisfied inclusion criteria and were maintained without modification (see Table 5). Consequently, primary indicators did not require a second round of expert consultation.

**TABLE 5:** First-round expert consultation results for primary indicators.

Primary indicators	Applicability				Importance				Operational feasibility			
	M	SD	CV	FR(%)	M	SD	CV	FR(%)	M	SD	CV	FR(%)
1 Structural indicator	4.88	0.34	0.07	87.50	4.81	0.54	0.11	87.50	4.81	0.54	0.11	87.50
2 Process indicator	4.88	0.34	0.07	87.50	5.00	0.00	0.00	100.00	4.88	0.50	0.10	93.75
3 Outcome indicator	4.81	0.54	0.11	87.50	4.88	0.34	0.07	87.50	4.81	0.54	0.11	87.50

M, mean; SD, standard deviation; CV, coefficient of variation; FR, full score rate.

Secondary indicators achieved consensus thresholds for appropriateness (mean = 4.75–4.94, CV = 0.05–0.14; full score rate = 87.50–93.75%) and operational feasibility (mean = 4.63–4.94, CV = 0.05–0.16; full score rate = 68.75–93.75%). Importance scores demonstrated greater variation (mean = 3.88–4.94, CV = 0.05–0.23), though 93.75% of items maintained full score rates of ≥ 31.25% (see Table 6). Despite broad consensus, experts recommended essential revisions to enhance clinical alignment: (1) modifying “1-h bundle management” to “Early Intervention”; (2) distinguishing non-invasive versus invasive monitoring in nursing surveillance protocols according to WHO risk stratification standards; (3) incorporating multidisciplinary collaboration metrics across structural and process domains. These significant modifications required second-round validation.

**TABLE 6:** Results of the first round of expert consultations on secondary indicators.

Secondary indicators	Applicability				Importance				Operational feasibility			
	M	SD	CV	FR(%)	M	SD	CV	FR(%)	M	SD	CV	FR(%)
1.1 Human resource allocation	4.81	0.54	0.11	87.50	4.06	0.93	0.23	43.75	4.63	0.72	0.16	75.00
1.2 Knowledge and skill training	4.94	0.25	0.05	93.75	4.88	0.34	0.07	87.50	4.94	0.25	0.05	93.75
2.1 Early identification of sepsis	4.81	0.54	0.11	87.50	4.94	0.25	0.05	93.75	4.63	0.72	0.16	75.00
2.2 1-h sepsis bundle care	4.75	0.68	0.14	87.50	3.88	0.89	0.23	31.25	4.75	0.68	0.14	87.50
2.3 Nursing monitoring techniques	4.88	0.50	0.10	93.75	4.94	0.25	0.05	93.75	4.63	0.72	0.16	75.00
3.1 Clinical outcomes	4.88	0.34	0.07	87.50	4.13	0.96	0.23	50.00	4.81	0.40	0.08	81.25
3.2 Nursing care quality	4.94	0.25	0.05	93.75	4.13	0.81	0.20	37.50	4.69	0.48	0.10	68.75

M, mean; SD, standard deviation; CV, coefficient of variation; FR, full score rate.

Among tertiary indicators, three items were excluded due to excessive variability (CV > 0.25): “SIRS assessment compliance” (2.1.1, all CV > 0.25), “nursing documentation accuracy” (3.2.1, CV = 0.29/0.28/0.26), and “health education awareness” (3.2.2, CV = 0.26-0.29; importance full score rate = 18.75%) (see Table 7). Retained items underwent evidence-based revisions including: (1) standardized terminology alignment with the Sepsis-3 international clinical guidelines, replacing SIRS with the more evidence-based qSOFA/SOFA as the core assessment tool for sepsis early identification; (2) expanded pathogen detection protocols; (3) implementation of restrictive fluid resuscitation with vasopressor protocols and fluid responsiveness testing; (4) addition of risk-adjusted 28-day sepsis mortality metrics to isolate non-nursing confounders. Multidisciplinary collaboration elements and nursing-sensitive outcome measures were systematically integrated. Following Study Group discussions, the expert modifications from the first round underwent revision, and adjusted indicators proceeded to second-round expert review and analysis.

**TABLE 7:** First-round expert consultation results for tertiary indicators.

Tertiary indicators	Applicability				Importance				Operational Feasibility			
	M	SD	CV	FR(%)	M	SD	CV	FR(%)	M	SD	CV	FR(%)
1.1.1 Patient-nurse ratio	4.81	0.40	0.08	81.25	4.81	0.40	0.08	81.25	4.56	0.63	0.14	62.50
1.1.2 Nurse academic qualification ratio	4.44	0.81	0.18	62.50	4.38	0.81	0.18	56.25	4.44	0.73	0.16	56.25
1.2.1 Achievement rate of training in relevant theoretical knowledge	4.94	0.25	0.05	93.75	4.44	0.73	0.16	56.25	4.94	0.25	0.05	93.75
1.2.2 Achievement rate of skills training in relevant specialties	5.00	0.00	0.00	100.00	4.94	0.25	0.05	93.75	5.00	0.00	0.00	100.00
2.1.1 SIRS assessment implementation rate	4.19	1.11	0.26*	62.50	3.88	1.15	0.30*	31.25	4.31	1.14	0.26*	68.75
2.1.2 qSOFA/SOFA assessment implementation rate	4.94	0.25	0.05	93.75	4.31	0.79	0.18	50.00	4.81	0.54	0.11	87.50
2.2.1 Implementation rate of the first lactate test	4.81	0.75	0.16	93.75	4.44	0.89	0.20	62.50	4.75	0.77	0.16	87.50
2.2.2 Implementation rate of two blood culture collections	4.81	0.40	0.08	81.25	4.50	0.63	0.14	56.25	4.50	1.03	0.23	75.00
2.2.3 Antibiotic use implementation rate	4.75	0.58	0.12	81.25	4.38	0.72	0.16	50.00	4.38	0.96	0.22	62.50
2.2.4 Liquid expansion implementation rate	4.75	0.58	0.12	81.25	4.38	0.81	0.18	56.25	4.63	1.02	0.22	87.50
2.2.5 Vasopressin implementation rate	4.56	0.81	0.18	75.00	4.25	0.86	0.20	50.00	4.19	0.98	0.23	50.00
2.3.1 Vital signs monitoring implementation rate	4.94	0.25	0.05	93.75	4.81	0.40	0.08	81.25	4.88	0.50	0.10	93.75
2.3.2 Implementation rate of urine output monitoring	4.94	0.25	0.05	93.75	4.75	0.45	0.09	75.00	4.88	0.50	0.10	93.75
2.3.3 MAP monitoring implementation rate	4.94	0.25	0.05	93.75	4.81	0.40	0.08	81.25	4.88	0.50	0.10	93.75
2.3.4 CVP monitoring implementation rate	4.75	0.58	0.12	81.25	4.50	0.73	0.16	62.50	4.44	0.89	0.20	62.50
2.3.5 ScvO_2_ monitoring implementation rate	4.69	0.60	0.13	75.00	4.44	0.73	0.16	56.25	4.81	0.40	0.08	81.25
2.3.6 Passive leg raising test execution rate	4.81	0.40	0.08	81.25	4.25	0.68	0.16	37.50	4.19	0.98	0.23	50.00
3.1.1 Incidence of septic shock	4.81	0.54	0.11	87.50	4.50	0.73	0.16	62.50	4.88	0.34	0.07	87.50
3.1.2 28-day hospitalization mortality rate	4.75	0.58	0.12	81.25	4.31	0.70	0.16	43.75	4.75	0.58	0.12	81.25
3.1.3 30-day readmission rate	4.75	0.68	0.14	87.50	4.38	0.72	0.16	50.00	4.88	0.50	0.10	93.75
3.2.1 Percentage of correctly written nursing documents	4.31	1.25	0.29*	75.00	4.25	1.18	0.28*	68.75	4.31	1.14	0.26*	68.75
3.2.2 Health promotion awareness rate	4.38	1.15	0.26*	75.00	3.63	0.96	0.26*	18.75*	4.25	1.24	0.29*	68.75
3.2.3 Nursing care satisfaction rate	4.81	0.40	0.08	81.25	4.75	0.77	0.16	87.50	4.88	0.34	0.07	87.50

*Does not meet the inclusion criteria. M, mean; SD, standard deviation; CV, coefficient of variation; FR, full score rate; MAP, Mean Arterial Pressure; CVP, Central Venous Pressure; ScvO_2_, Central Venous Oxygen Saturation; SIRS, Systemic Inflammatory Response Syndrome; SOFA, Sequential Organ Failure Assessment; qSOFA, Quick Sequential Organ Failure Assessment.

### Second round Delphi results

The second Delphi round validated the finalized sepsis care-sensitive quality indicator system, comprising 3 primary, 9 secondary, and 30 tertiary indicators. Secondary indicators demonstrated high consensus across all domains, with appropriateness scores ranging from 3.94 to 4.75 (coefficient of variation [CV] = 0.12–0.24; full score rate = 37.50–81.25%). Importance ratings showed stronger agreement (mean = 4.25–4.75; CV = 0.07–0.16; full score rate = 37.50–75.00%), while operational feasibility scores remained consistent (mean = 4.25–4.69; CV = 0.13–0.16; full score rate = 37.50–75.00%). All secondary indicators met predefined inclusion criteria without further revisions (see Table 8).

**TABLE 8:** First-round expert consultation results for secondary indicators.

Secondary indicators	Applicability				Importance				Operational feasibility			
	M	SD	CV	FR(%)	M	SD	CV	FR(%)	M	SD	CV	FR(%)
1.1 Human resource allocation	3.94	0.93	0.24	37.50	4.69	0.48	0.10	68.75	4.56	0.73	0.16	68.75
1.2 Knowledge and skill training	4.56	0.73	0.16	68.75	4.75	0.45	0.09	75.00	4.50	0.73	0.16	62.50
1.3 Multidisciplinary teamwork	4.75	0.58	0.12	81.25	4.56	0.73	0.16	68.75	4.69	0.60	0.13	75.00
2.1 Early identification	4.31	0.60	0.14	37.50	4.44	0.81	0.18	62.50	4.50	0.63	0.14	56.25
2.2 Early intervention	4.13	0.81	0.20	37.50	4.50	0.73	0.16	62.50	4.31	0.79	0.18	50.00
2.3 Monitoring techniques	4.63	0.62	0.13	68.75	4.88	0.34	0.07	87.50	4.69	0.60	0.13	75.00
2.4 Multidisciplinary Collaboration	4.75	0.58	0.12	81.25	4.25	0.68	0.16	37.50	4.50	0.63	0.14	56.25
3.1 Clinical outcomes	4.19	0.83	0.20	43.75	4.25	0.68	0.16	37.50	4.25	0.68	0.16	37.50
3.2 Quality of care	4.69	0.60	0.13	75.00	4.69	0.48	0.10	68.75	4.63	0.72	0.16	75.00

For tertiary indicators, substantial consensus emerged: appropriateness (mean = 4.13–4.88; CV = 0.07–0.20; full score rate = 31.25–87.50%), importance (mean = 4.06–4.75; CV = 0.09–0.20; full score rate = 31.25–75.00%), and operational feasibility (mean = 4.13–4.94; CV = 0.05–0.20; full score rate = 31.25–93.75%). No tertiary indicators required elimination or modification, confirming their clinical relevance and measurability (see Table 9). Experts proposed no additional revisions in this round, demonstrating the framework’s stability.

**TABLE 9:** Second-round expert consultation results for tertiary indicators.

Tertiary indicators	Applicability				Importance				Operational feasibility			
	M	SD	CV	FR(%)	M	SD	CV	FR(%)	M	SD	CV	FR(%)
1.1.1 Nurse-to-bed ratio	4.56	0.63	0.14	62.50	4.44	0.81	0.18	62.50	4.31	0.79	0.18	50.00
1.1.2 Ratio of nurses’ academic qualifications	4.50	0.63	0.14	56.25	4.06	0.77	0.19	31.25	4.31	0.79	0.18	50.00
1.1.3 Nurse work experience ratio	4.56	0.63	0.14	62.50	4.69	0.48	0.10	68.75	4.19	0.83	0.20	43.75
1.2.1 Achievement rate of training in relevant theoretical knowledge	4.56	0.63	0.14	62.50	4.44	0.73	0.16	56.25	4.69	0.60	0.13	75.00
1.2.2 Achievement rate of skills training in relevant specialties	4.75	0.58	0.12	81.25	4.06	0.77	0.19	31.25	4.69	0.60	0.13	75.00
1.3.1 Completeness of core unit configuration	4.63	0.62	0.13	68.75	4.13	0.72	0.17	31.25	4.56	0.51	0.11	56.25
1.3.2 Adherence to sepsis knowledge training	4.63	0.62	0.13	68.75	4.63	0.62	0.13	68.75	4.75	0.58	0.12	81.25
2.1.1 Completion rate of early recognition assessment	4.75	0.58	0.12	81.25	4.75	0.45	0.09	75.00	4.44	0.51	0.12	43.75
2.1.2 Dynamic assessment of adherence rates	4.31	0.60	0.14	37.50	4.31	0.60	0.14	37.50	4.25	0.86	0.20	50.00
2.2.1 Rate of timely opening of intravenous access	4.38	0.89	0.20	62.50	4.63	0.62	0.13	68.75	4.50	0.73	0.16	62.50
2.2.2 Implementation rate of the first lactate test	4.69	0.60	0.13	75.00	4.31	0.70	0.16	43.75	4.81	0.40	0.08	81.25
2.2.3 Antimicrobial administration before pathogen identification rate	4.69	0.60	0.13	75.00	4.44	0.81	0.18	62.50	4.88	0.34	0.07	87.50
2.2.4 Antibiotic use implementation rate	4.56	0.63	0.14	62.50	4.50	0.73	0.16	62.50	4.69	0.60	0.13	75.00
2.2.5 Restricted fluid resuscitation implementation rate	4.25	0.68	0.16	37.50	4.25	0.77	0.18	43.75	4.25	0.68	0.16	37.50
2.2.6 Vasoactive drug implementation rate	4.50	0.73	0.16	62.50	4.06	0.77	0.19	31.25	4.50	0.73	0.16	62.50
2.3.1 Non-invasive monitoring: implementation rate of vital signs monitoring	4.38	0.81	0.18	56.25	4.69	0.48	0.10	68.75	4.88	0.34	0.07	87.50
2.3.2 Non-invasive/invasive monitoring: implementation rate of urine output monitoring	4.75	0.45	0.09	75.00	4.50	0.63	0.14	56.25	4.63	0.62	0.13	68.75
2.3.3 Non-invasive monitoring: MAP monitoring implementation rate	4.69	0.60	0.13	75.00	4.44	0.81	0.18	62.50	4.88	0.34	0.07	87.50
2.3.4 Invasive monitoring: CVP monitoring normative implementation rate	4.69	0.60	0.13	75.00	4.38	0.81	0.18	56.25	4.31	0.70	0.16	43.75
2.3.5 Invasive monitoring: normative implementation rate of ScvO_2_ monitoring	4.69	0.60	0.13	75.00	4.31	0.79	0.18	50.00	4.38	0.72	0.16	50.00
2.3.6 Rate of implementation of rehydration test norms	4.13	0.72	0.17	31.25	4.06	0.77	0.19	31.25	4.25	0.68	0.16	37.50
2.4.1 Implementation rate of MDT consultations	4.69	0.60	0.13	75.00	4.13	0.72	0.17	31.25	4.88	0.34	0.07	87.50
2.4.2 Implementation rate of joint multidisciplinary rounds	4.69	0.60	0.13	75.00	4.56	0.73	0.16	68.75	4.13	0.81	0.20	37.50
2.4.3 Standardized communication tool usage rate	4.38	0.62	0.14	43.75	4.19	0.75	0.18	37.50	4.94	0.25	0.05	93.75
3.1.1 Septic shock incidence	4.56	0.63	0.14	62.50	4.50	0.73	0.16	62.50	4.31	0.60	0.14	37.50
3.1.2 Risk-adjusted 28-day mortality	4.50	0.73	0.16	62.50	4.19	0.75	0.18	37.50	4.38	0.62	0.14	43.75
3.1.3 30-day infection-related readmission rate	4.31	0.87	0.20	56.25	4.31	0.87	0.20	56.25	4.13	0.72	0.17	31.25
3.2.1 The incidence of catheter-related bloodstream infections	4.63	0.62	0.13	68.75	4.38	0.72	0.16	50.00	4.50	0.73	0.16	62.50
3.2.2 The incidence of pressure ulcers	4.63	0.62	0.13	68.75	4.19	0.83	0.20	43.75	4.63	0.62	0.13	68.75
3.2.3 Patient satisfaction with nursing care	4.88	0.34	0.07	87.50	4.56	0.73	0.16	68.75	4.56	0.63	0.14	62.50

MAP, Mean Arterial Pressure; CVP, Central Venous Pressure; ScvO_2_, Central Venous Oxygen Saturation; SIRS, Systemic Inflammatory Response Syndrome; SOFA, Sequential Organ Failure Assessment; qSOFA, Quick Sequential Organ Failure Assessment; MDT, multidisciplinary team.

The finalized system establishes a hierarchical structure comprising three primary domains, nine secondary domains, and thirty actionable tertiary indicators, creating a validated tool for assessing nursing care quality in sepsis management (see Supplementary File 1). The weights and combination weights for the sepsis care quality sensitive indicator evaluation system are detailed in Table 10. This two-round Delphi process achieved unanimous expert consensus, confirming the system’s alignment with clinical guidelines and practical feasibility.

**TABLE 10:** Weighting system for sensitive indicators of sepsis care.

Primary indicators	Primary weights	Secondary indicators	Secondary weights	Combined weights	Tertiary indicators	Tertiary weights	Combined weights
Structural indicators	0.3119	1.1 Human resource allocation	0.3119	0.0973	1.1.1	0.3202	0.0312
					1.1.2	0.1226	0.0119
					1.1.3	0.5571	0.0542
		1.2 Knowledge and skill training	0.4905	0.1530	1.2.1	0.7500	0.1147
					1.2.2	0.2500	0.0382
		1.3 Multidisciplinary Teamwork	0.1976	0.0616	1.3.1	0.2500	0.0154
					1.3.2	0.7500	0.0462
Process indicators	0.4905	2.1 Early identification	0.1625	0.0797	2.1.1	0.7500	0.0598
					2.1.2	0.2500	0.0199
		2.2 Early intervention	0.2260	0.1108	2.2.1	0.3264	0.0362
					2.2.2	0.1219	0.0135
					2.2.3	0.1742	0.0193
					2.2.4	0.2178	0.0241
					2.2.5	0.0968	0.0107
					2.2.6	0.0629	0.0070
		2.3 Monitoring techniques	0.5069	0.2486	2.3.1	0.3246	0.0807
					2.3.2	0.2164	0.0538
					2.3.3	0.1729	0.0430
					2.3.4	0.1313	0.0326
					2.3.5	0.0956	0.0238
					2.3.6	0.0592	0.0147
		2.4 Multidisciplinary collaborative processes	0.1046	0.0513	2.4.1	0.1593	0.0082
					2.4.2	0.5889	0.0302
					2.4.3	0.2519	0.0129
Outcome indicators	0.1976	3.1 Clinical outcomes	0.2500	0.0494	3.1.1	0.5390	0.0266
					3.1.2	0.1638	0.0081
					3.1.3	0.2973	0.0147
		3.2 Quality of care	0.7500	0.1482	3.2.1	0.2973	0.0441
					3.2.2	0.1638	0.0243
					3.2.3	0.5390	0.0799

## Discussion

This Delphi study developed a three-tiered sepsis care quality indicator system, consisting of 3 primary, 9 secondary, and 30 tertiary indicators, based on Donabedian’s Structure-Process-Outcome framework. The slight decrease and plateau of Kendall’s W coordination coefficient in the second Delphi round relative to the first is a reasonable phenomenon, attributed to the refined and more specific tertiary indicator items in the second round: experts held evidence-based clinical divergences on the operational feasibility and rationality of these detailed tertiary indicators, which is a normal reflection of in-depth professional discussion rather than a lack of consensus; all coefficients still remained significant (*P* < 0.05), indicating the overall expert consensus on the indicator system was still robust and reliable. The hierarchical weight distribution—process indicators (weight = 0.491) > structural indicators (0.312) > outcome indicators (0.197)—reflects the time-sensitive nature of sepsis management, where frontline clinical interventions supersede structural prerequisites in determining patient outcomes. This corresponds with evidence linking hourly delays in sepsis treatment to a 7.6% increase in mortality, emphasizing the critical role of nurses in rapid decision-making (39, 40).

Within structural domains, the secondary indicator hierarchy (knowledge-skill training > human resources > multidisciplinary configuration) reveals significant gaps in sepsis education and workforce optimization. The highest-weighted tertiary structural indicator, “*1.2.1 Theoretical knowledge compliance rate*” (weight = 0.1147), demonstrates the significant impact of standardized training programs, which increase 1-h bundle adherence from 29 to 63% (41). However, three key challenges emerge: (1) Competency deteriorates at a rate of 31% per 6 months without refresher training (42), necessitating microlearning interventions. (2) Advanced practice nurses with 10 or more years of experience exhibited superior knowledge of sepsis and improved recognition and intervention rates (43, 44). Yet, their reluctance to adopt evidence-based strategies indicates a tension between experience and adaptability (45). (3) Although healthcare organizations meet basic requirements for multidisciplinary teams, few achieve effective operational integration (17). Evidence suggests that meeting administrative compliance standards alone fails to ensure quality care coordination (46).

Among the optimized process indicators, monitoring technology’s high weight (0.2486) emphasizes its pivotal role in sepsis management, with its tertiary indicator “implementation rate of non-invasive vital signs monitoring” ranking second, aligning with WHO’s advocacy for non-invasive technology (47) and the “golden hour” intervention principle (15, 48). However, this weighting may oversimplify sepsis management complexities and overlook technology dependence risks. While standardized vital sign monitoring reduces ICU transfers (17), high weightings might mask resource disparities, such as “inflated implementation rates” due to limited invasive monitoring availability in primary care facilities (49). Furthermore, emphasizing monitoring implementation rates over data interpretation and clinical response may diminish nurses’ decision-making role (13).

The weight difference between early intervention and early identification (0.1108 vs. 0.0797) reflects operational feasibility priorities. However, the low weighting of venous access contrasts with its critical role in “golden hour” interventions, despite evidence supporting expanded venous access team functions (17). Early identification tools (11), including Quick Sequential Organ Failure Assessment (qSOFA), SIRS criteria, and NEWS, are widely recommended for rapid screening (12, 13). While qSOFA/SAFA was selected based on literature and expert consensus (11), its sensitivity remains debatable compared to EWS and SIRS (49), potentially limiting early recognition, particularly in non-ICU settings (12).

The relatively low weighting of multidisciplinary collaborative processes (0.0513) contradicts their demonstrated value, indicating a metrics system bias toward technical procedures over teamwork. Research demonstrates that multidisciplinary collaboration effectively reduces antibiotic misuse and accelerates diagnosis (9, 17). The limited representation of nursing specialists on the expert panel may have diminished their influence in multidisciplinary decision-making processes (50).

Within the sepsis care quality framework, outcome indicators encompass clinical outcomes and care quality dimensions. The tertiary indicator “*3.2.1 Nursing satisfaction rate*” (weight = 0.0799) emerged as predominant, challenging traditional assumptions about patient experience metrics lacking clinical relevance by demonstrating a direct correlation between nurse engagement and protocol adherence.

The second-ranked indicator, “*3.2.1 Incidence of catheter-related bloodstream infections*” (weight = 0.0441), reflects established care bundles. This weighting demonstrates that standardized insertion and maintenance protocols have reduced variability across high-performing institutions (40). The Delphi panel discussions revealed professional perspectives: nursing experts noted that this metric encompasses medical factors, including antibiotic stewardship, while physicians emphasized nursing’s essential role in aseptic compliance. This divergence highlights the importance of interdisciplinary ownership of infection control metrics.

Paradoxically, “*3.1.2 Risk-adjusted 28-day mortality*” (weight = 0.0081) received low scores despite methodological improvements to isolate nursing-sensitive outcomes. The risk-adjustment model incorporated disease severity (APACHE III), comorbidities (Charlson Index), and resource utilization variables; however, confounding factors remain. Nursing panelists maintained that medical decisions, such as vasopressor titration significantly influence mortality. Conversely, physicians emphasized nursing’s crucial role in detecting early deterioration, a key determinant of survival in sepsis (51).

This 30-item sepsis care quality indicator system features strong practical implementability in clinical settings, as its indicators are highly compatible with mainstream hospital information systems (HIS) and electronic health records (EHR) in China. Most quantitative indicators (e.g., monitoring implementation rates, incidence of adverse events) can be integrated into HIS/EHR for automated data extraction and real-time statistical analysis, which effectively minimizes the manual documentation and data collection burden on frontline nurses in EDs and ICUs with high workloads. For individual qualitative indicators requiring standardized evaluation, simple embedded assessment modules can be added to EHR to realize semi-automated data collection, balancing the accuracy of indicator statistics and the convenience of clinical implementation. In addition, the hierarchical weight design of the system allows medical institutions to conduct targeted quality improvement based on indicator importance, and flexibly adjust the data collection focus according to their own HIS construction level and clinical needs.

## Limitations

While this study presents a validated framework for evaluating sepsis care quality, several limitations merit consideration. First, the Delphi panel consisted exclusively of experts from tertiary hospitals in China, potentially limiting the generalizability of the findings to resource-constrained settings, such as community hospitals. Second, excluding primary care clinicians and nurses introduces selection bias, as their perspectives on care barriers remain unexplored. Third, the constructivist paradigm inherently favors expert consensus over predictive validity testing, necessitating future empirical validation of the relationships between indicators and outcomes. Finally, cultural and systemic differences in healthcare delivery may restrict the direct applicability of these NSQIs to non-Chinese contexts, particularly in settings with different nursing scopes of practice or infection control protocols.

## Conclusion

This study established China’s first sepsis-specific nursing-sensitive quality indicator system through a comprehensive mixed-methods approach, combining Donabedian’s framework with frontline clinical insights. The hierarchical system (3 primary, 9 secondary, 30 tertiary indicators) prioritizes process-driven interventions, highlighting nurses’ vital role in time-sensitive sepsis management. Future research should validate these indicators across diverse healthcare settings, examine their predictive validity for patient outcomes, and adapt the framework through cross-cultural comparisons. This tool can standardize sepsis care quality assessments and inform policy reforms in China and comparable healthcare systems by connecting evidence-based guidelines with measurable nursing contributions.

## Data Availability

The original contributions presented in the study are included in the article/Supplementary material, further inquiries can be directed to the corresponding author.
