# Supplementary material for: Construction of a sensitive indicator evaluation system for sepsis care quality: a three-stage mixed-method Delphi study
Source: Front Med (Lausanne). 2026 May 4;13:1815560. doi: 10.3389/fmed.2026.1815560 (PMC13180948; doi:10.3389/fmed.2026.1815560)
Supplement: Supplementary file 1 [file Data_Sheet_1.pdf]

Table 10 Construction of Sensitive Indicators

Primary indicators	Secondary indicators	Tertiary indicators	Dimension		Significance	Formula for calculation	Interpretation	Data collection method
			Essential	Selectable				
1. Structural indicators	1.1 Human resource allocation	1. 1. 1 nurse-to-bed ratio	√		Reflects the allocation of human resources.	$\frac{Total\ number\ of\ nurses}{Actual\ number\ of\ beds\ opened}$	Nurses are defined as registered, full-time staff within the healthcare facility.	Electronic Human Resources Management System
		1. 1. 2 Ratio of nurses' academic qualifications	√		Reflects the educational structure of the nursing team.	$\frac{Number\ of\ nurses\ with\ a\ certain\ level\ of\ education}{Total\ number\ of\ nurses}$	Education levels include secondary, college, bachelor's, master's, and doctoral degrees (based on official academic transcripts).	Electronic Human Resources Management System
		1.1.3 Ratio of nurses' working years	√		Reflects the structure of the nursing team in terms of years of experience.	$\frac{Number\ of\ nurses\ with\ a\ certain\ number\ of\ years\ of\ service}{Total\ number\ of\ nurses}$	Experience is defined as cumulative years working as a registered nurse, categorized as ≤5 years, 5–10 years, and >10 years.	Electronic Human Resources Management System
	1. 2 Knowledge and skill training	1. 2. 1 Achievement rate of training in relevant theoretical knowledge	√		Reacting to the implementation of nursing theory training.	$\frac{Number\ of\ nurses\ who\ met\ the\ theoretical\ knowledge\ test}{Total\ number\ of\ nurses} \times 100\%$	Training includes sepsis definition updates, clinical guidelines (e.g., 2021 Surviving Sepsis Campaign), manifestations, and standardized nursing protocols. Passing criteria: ≥ 80% score on departmental written exam.	Unit Training & Assessment Records
		1. 2. 2 Achievement rate of skills training in relevant specialties	√		Reacting to the implementation of nursing skills training.	$\frac{Number\ of\ nurses\ trained\ to\ standard\ in\ specialized\ skills}{Total\ number\ of\ nurses} \times 100\%$	Target skills: blood culture collection, CVP/$ScvO_2$ monitoring, ventilator operation, CRRT, ultrasound-guided procedures, and arterial blood pressure management. Passing criteria: ≥90% score on hands-on competency assessment.	Unit Training & Assessment Records
	1. 3 Multidisciplina	1. 3. 1 Completeness	√		Reflects the structural support capacity of the	$\frac{Actual\ number\ of\ core\ sections\ configured}{Number\ of\ core\ sections\ required\ by\ the\ guidelines} \times 100\%$	Core departments per 2021 Surviving Sepsis Campaign: Emergency Department, ICU,	Hospital sepsis MDT configuration registry

	ry teamwork indicator	of core unit configuration		multidisciplinary team.		Clinical Pharmacy, and Infectious diseases specialty.	
		1. 3. 2 Adherence to sepsis knowledge training	√	Reflects the level of capacity building of the team.	$\frac{Number\ of\ people\ who\ have\ reached\ the\ standard\ in\ the\ assessment}{Number\ of\ multidisciplinary\ team\ members} \times 100\%$	Aligns with WHO Patient Safety Guidelines: ⩾90% annual participation in sepsis-specific interprofessional training (including knowledge and simulation-based assessments).	MDT Training Attendance & Assessment Logs
2 Process indicators	2. 1 Early identification	2. 1. 1 Completion rate of early recognition assessment	√	Reflects the ability of healthcare organizations to identify early and standardize care for patients at high risk for sepsis.	$\frac{Number\ of\ patients\ with\ suspected\ infection\ with\ early\ assessment\ completed\ within\ 1h}{Total\ number\ of\ patients\ with\ suspected\ infection\ in\ the\ same\ period} \times 100\%$	Suspected infection is defined by clinical documentation (e.g., "rule out sepsis" or "infection suspected"). Compliance with 2021 Surviving Sepsis Campaign recommendation for rapid risk stratification.	Electronic Health Records (EHR)
		2. 1. 2 Dynamic assessment of adherence rates	√	Reflects the standardization of continuity of care and dynamic monitoring of patients with sepsis.	$\frac{Number\ of\ actual\ completions\ eligible\ for\ repeat\ assessments}{Total\ number\ of\ repeat\ assessments\ to\ be\ completed} \times 100\%$	Target population: patients with SOFA ⩾2 or qSOFA ⩾2. Assessments include repeat SOFA/qSOFA to monitor clinical deterioration.	EHR automated assessment tracking
	2. 2 Early intervention	2. 2. 1 Rate of timely intravenous access establishment	√	Reflects the timeliness of opening veins in patients with suspected or confirmed sepsis.	$\frac{Number\ of\ patients\ at\ high\ risk\ of\ suspected\ or\ confirmed\ sepsis\ with\ open\ veins\ within\ 15\ min}{Total\ number\ of\ patients\ with\ suspected\ or\ confirmed\ sepsis\ during\ the\ same\ period} \times 100\%$	V access defined as ⩾ 20G peripheral or central venous catheter placement, per WHO and NICE guidelines (NG51).	EHR medication administration logs
		2. 2. 2 Implementation rate of initial lactate	√	Timeliness and standardization of reactive lactate monitoring.	$\frac{Number\ of\ patients\ with\ suspected\ or\ confirmed\ sepsis\ with\ lactate\ testing\ completed\ within\ 1h}{Total\ number\ of\ patients\ with\ suspected\ or\ confirmed\ sepsis\ during\ the\ same\ period} \times 100\%$	Includes order placement, blood collection, laboratory processing, and result availability. Positive sepsis diagnosis: SOFA ⩾2 or qSOFA ⩾2 with clinical suspicion.	EHR laboratory results interface

	measurement						
	2.2.3 The rate of delivering antimicrobial drugs before the treatment of pathogens	√		Reacting to the standardization of pathogenetic delivery before antimicrobial drug use.	$\frac{\text{Number of patients completing pathogenetic sentinel testing prior to antimicrobial use}}{\text{Total number of patients treated with antimicrobial drugs during the same period}} \times 100\%$	Specimens include blood, sputum, urine, and drainage cultures; molecular testing (e.g., next-generation sequencing).	EHR microbiology order/collection records
	2.2.4 Antibiotic administration implementation rate	√		Reflecting the standardization of early broad-spectrum antimicrobial drug use in patients with sepsis.	$\frac{\text{Number of patients with suspected or confirmed sepsis starting antibiotics within } 1h}{\text{Total number of patients with suspected or sepsis during the same period}} \times 100\%$	Includes physician order verification, medication preparation, and intravenous administration. Confirmed sepsis: documented SOFA ≥2 or qSOFA ≥2 with provider diagnosis.	EHR medication administration timeline
	2.2.5 Controlled fluid resuscitation implementation rate		√	Reflecting the standardization and timeliness of restrictive fluid resuscitation in patients with septic shock.	$\frac{\text{Number of patients performing restrictive fluid resuscitation within } 1h}{\text{Total number of patients with septic shock during the same period}} \times 100\%$	Restrictive protocol: cautious fluid administration guided by hemodynamic monitoring (e.g., CVP, $ScvO_2$), per 2021 Surviving Sepsis Campaign.	EHR fluid balance and order records
	2.2.6 Vasoactive medication implementation rate		√	Reflecting standardization and timeliness of vasoactive drug use in patients with septic shock.	$\frac{\text{Number of patients eligible and initiating vasoactive drugs within } 1h}{\text{Total number of eligible septic shock patients during the same period}} \times 100\%$	Eligibility: MAP <65 mmHg despite fluid resuscitation or lactate ≥2 mmol/L. Includes norepinephrine/epinephrine initiation per guideline-recommended protocols.	EHR hemodynamic monitoring records
2.3 Monitoring techniques	2.3.1 Non-invasive monitoring: implementatio	√		Reflects the continuity and standardization of vital sign monitoring in patients with sepsis.	$\frac{\text{Number of sepsis patients with correctly performed vital signs monitoring}}{\text{Total number of patients with sepsis during the same period}} \times 100\%$	Includes continuous monitoring of heart rate, blood pressure, respiratory rate, and oxygen saturation, with hourly EHR documentation.	EHR vital signs monitoring module

	n rate of vital signs monitoring						
	2. 3. 2 Non-invasive/invasive monitoring: implementation rate of urine output monitoring	√		Reflecting continuity and standardization of urine output monitoring in patients with sepsis.	$\frac{Number\ of\ patients\ with\ sepsis\ who\ correctly\ perform\ urine\ output\ monitoring}{Total\ number\ of\ patients\ with\ sepsis\ during\ the\ same\ period} \times 100\%$	Aligns with Sepsis-6 bundle: critical for early detection of renal hypoperfusion.	EHR intake/output documentation
	2. 3. 3 Non-invasive monitoring: mean arterial pressure (MAP) monitoring implementation rate	√		Reflects the standardization of MAP monitoring in patients with sepsis.	$\frac{Number\ of\ sepsis\ patients\ with\ correctly\ performed\ MAP\ monitoring}{Total\ number\ of\ patients\ with\ sepsis\ during\ the\ same\ period} \times 100\%$	MAP is a core hemodynamic target per 2021 Surviving Sepsis Campaign; reflects adequacy of organ perfusion.	EHR hemodynamic parameter records
	2. 3. 4 Invasive monitoring: central venous pressure (CVP) monitoring normative		√	Reflecting the standardization of early hemodynamic monitoring in patients with septic shock.	$\frac{Number\ of\ patients\ with\ sepsis\ for\ whom\ CVP\ monitoring\ is\ standardized}{Total\ number\ of\ patients\ with\ sepsis\ who\ met\ the\ indications\ for\ CVP\ monitoring\ during\ the\ same\ period} \times 100\%$	Indications: septic shock or lactate ⩾ 4 mmol/L. Normative practice includes proper catheter insertion, zeroing, and interpretive documentation.	EHR invasive monitoring procedure logs

	implementation rate						
	2.3.5 Invasive monitoring: central venous oxygen saturation (ScvO2) monitoring protocol implementation rate		√	Reflecting the normality of dynamic assessment of tissue perfusion in patients with septic shock.	$\frac{Number\ of\ patients\ with\ sepsis\ for\ whom\ \text{ScvO2}\ monitoring\ is\ standardized}{Total\ number\ of\ patients\ with\ sepsis\ who\ met\ the\ indications\ for\ ScvO2\ monitoring\ during\ the\ same\ period} \times 100\%$	Indications: fluid-refractory shock (MAP <65 mmHg or vasopressor-dependent) or persistent lactate ≥4 mmol/L. Normative values: 70%–80%; includes proper catheter placement and interpretive analysis.	EHR invasive monitoring procedure logs
	2.3.6 Rate of implementation of rehydration test norms		√	Reflecting the standardization of fluid management in patients with septic shock.	$\frac{Number\ of\ patients\ with\ sepsis\ for\ whom\ rehydration\ tests\ are\ performed\ on\ a\ standardized\ basis}{Total\ number\ of\ patients\ with\ sepsis\ who\ met\ the\ indications\ for\ rehydration\ testing\ during\ the\ same\ period} \times 100\%$	Indications: tissue hypoperfusion without overt shock. Normative practice: 500 mL crystalloid over 30 minutes with pre/post hemodynamic assessment (e.g., stroke volume variation).	EHR fluid challenge procedure records
2.4 Multidisciplinary collaborative processes	2.4.1 Implementation rate of multidisciplinary team (MDT) consultations	√		Reflects early teamwork and responsiveness.	$\frac{Number\ of\ cases\ in\ which\ MDT\ was\ initiated\ within\ 1h\ of\ diagnosis\ of\ sepsis}{Total\ number\ of\ patients\ with\ sepsis\ during\ the\ same\ period} \times 100\%$	Core MDT members: emergency, ICU, infectious diseases physicians, and clinical pharmacists, per 2021 Surviving Sepsis Campaign.	EHR consultation request timestamps
	2.4.2 Implementation rate of joint	√		Reflect the dynamic assessment of synergies.	$\frac{Actual\ number\ of\ days\ of\ joint\ room\ inspections\ performed}{Actual\ days\ of\ hospitalization\ days} \times 100\%$	Rounds include ≥2 disciplines (emergency/ICU/infectious diseases/clinical pharmacy/charge nurse). Frequency: daily in	On-site observer documentation

		multidisciplinary rounds				critical phase, 3×/week in stable phase (per 2023 ICU MDT Consensus).	
		2. 4. 3 Standardized communication tool utilization rate	√	Safeguard the integrity and continuity of key information in the communication process.	$\frac{Number\ of\ shifts\ with\ complete\ handover\ using\ SBAR}{Total\ number\ of\ shift\ handovers} \times 100\%$	SBAR components: Situation, Background, Assessment, Recommendation, per WHO Patient Safety Guidelines.	Direct observation of shift handover
3 Outcome indicators	3. 1 Clinical outcomes	3. 1. 1 Septic shock incidence	√	Quantitative assessment of the efficacy of quality of care in blocking critical illness conversion.	$\frac{Number\ of\ patients\ experiencing\ septic\ shock}{Total\ number\ of\ patients\ with\ sepsis\ during\ the\ same\ period} \times 100\%$	Septic shock defined as vasopressor-dependent hypotension (MAP <65 mmHg) and/or persistent lactate ≥2 mmol/L despite fluid resuscitation (2021 Surviving Sepsis Campaign).	EHR diagnostic coding and physician notes
		3. 1. 2 Risk-adjusted 28-day mortality	√	Accurately expose shortcomings from the quality of care by stripping away non-nursing factors that interfere.	$\frac{Number\ of\ sepsis-related\ deaths\ within\ 28\ days\ of\ hospital\ admission}{Total\ number\ of\ sepsis\ patients\ in\ the\ same\ period} \times 100\%$ $Standardized\ mortality\ ratio = \frac{Actual\ mortality\ rate}{Projected\ mortality} \times 100\%$	Risk adjustment factors: baseline SOFA score, Charlson Comorbidity Index, and nurse-to-bed ratio. Predicted mortality derived from validated models (e.g., APACHE II).	EHR mortality and comorbidity records
		3. 1. 3 30-day infection-related readmission rate	√	Reflects continuity of care and effectiveness of infection control after hospitalization.	$\frac{Number\ of\ patients\ with\ infection-related\ unplanned\ readmissions\ for\ sepsis}{Total\ number\ of\ sepsis\ patients\ discharged\ during\ the\ same\ period} \times 100\%$	Indications: sepsis recurrence, catheter-related bloodstream infection, surgical site infection (IDSA 2023 guidelines).	EHR readmission diagnosis records
	3. 2 Quality of care	3. 2. 1 The incidence of catheter-related bloodstream	√	Reflects the standardization of nursing practice and the effectiveness of preventive measures.	$\frac{Number\ of\ patients\ with\ sepsis\ who\ developed\ catheter-related\ bloodstream\ infections}{Total\ number\ of\ sepsis\ patients\ in\ the\ same\ period} \times 100\%$	CRBSI defined as positive blood culture matching catheter tip culture, per CDC NHSN criteria.	EHR infection control surveillance logs

	infections					
	3. 2. 2 The incidence of pressure ulcers	√	Reflects the standardization of nursing practice and the effectiveness of preventive measures.	$\frac{Number\ of\ septic\ patients\ with\ pressure\ ulcers}{Total\ number\ of\ sepsis\ patients\ in\ the\ same\ period} \times 100\%$	Staging per NPUAP 2016 guidelines; includes assessment frequency, repositioning, and nutritional support documentation.	EHR wound care documentation
	3. 2. 3 Patient satisfaction with nursing care	√	Reflects the overall quality of nursing services in terms of humanistic care, communication efficacy and technical operation.	$\frac{Actual\ score\ on\ satisfaction\ questionnaire}{Total\ satisfaction\ questionnaire\ score} \times 100\%$	Standardized inpatient satisfaction survey (e.g., Press Ganey), focusing on sepsis-specific care elements (e.g., explanation of treatment, timely response to needs).	Hospital Patient Satisfaction Survey System
